# Pancreatic Aquaporin-7: A Novel Target for Anti-diabetic Drugs?

**DOI:** 10.3389/fchem.2018.00099

**Published:** 2018-04-05

**Authors:** Leire Méndez-Giménez, Silvia Ezquerro, Inês V. da Silva, Graça Soveral, Gema Frühbeck, Amaia Rodríguez

**Affiliations:** ^1^Metabolic Research Laboratory, University of Navarra, Pamplona, Spain; ^2^CIBER Fisiopatología de la Obesidad y Nutrición, Instituto de Salud Carlos III, Madrid, Spain; ^3^Faculty of Pharmacy, Research Institute for Medicines (iMed.ULisboa), Universidade de Lisboa, Lisboa, Portugal; ^4^Department of Endocrinology and Nutrition, Clínica Universidad de Navarra, Pamplona, Spain

**Keywords:** aquaporin, glycerol, pancreas, insulin signaling, obesity, type 2 diabetes, bariatric surgery

## Abstract

Aquaporins comprise a family of 13 members of water channels (AQP0-12) that facilitate a rapid transport of water across cell membranes. In some cases, these pores are also permeated by small solutes, particularly glycerol, urea or nitric oxide, among other solutes. Several aquaporins have been identified in the pancreas, an exocrine and endocrine organ that plays an essential role in the onset of insulin resistance and type 2 diabetes. The exocrine pancreas, which accounts for 90% of the total pancreas, secretes daily large volumes of a near-isotonic fluid containing digestive enzymes into the duodenum. AQP1, AQP5, and AQP8 contribute to fluid secretion especially from ductal cells, whereas AQP12 allows the proper maturation and exocytosis of secretory granules in acinar cells of the exocrine pancreas. The endocrine pancreas (10% of the total pancreatic cells) is composed by the islets of Langerhans, which are distributed in α, β, δ, ε, and pancreatic polypeptide (PP) cells that secrete glucagon, insulin, somatostatin, ghrelin and PP, respectively. AQP7, an aquaglyceroporin permeated by water and glycerol, is expressed in pancreatic β-cells and murine studies have confirmed its participation in insulin secretion, triacylglycerol synthesis and proliferation of these endocrine cells. In this regard, transgenic AQP7-knockout mice develop adult-onset obesity, hyperinsulinemia, increased intracellular triacylglycerol content and reduced β-cell mass in Langerhans islets. Moreover, we have recently reported that AQP7 upregulation in β-cells after bariatric surgery, an effective weight loss surgical procedure, contributes, in part, to the improvement of pancreatic steatosis and insulin secretion through the increase of intracytoplasmic glycerol in obese rats. Human studies remain scarce and controversial, with some rare cases of loss-of function mutations of the *AQP7* gene being associated with the onset of type 2 diabetes. The present Review is focused on the role of aquaporins in the physiology and pathophysiology of the pancreas, highlighting the role of pancreatic AQP7 as a novel player in the control of β-cell function and a potential anti-diabetic-drug.

## Introduction

The movement of water through the lipid bilayers of cell membranes is essential for homeostasis. Aquaporins (AQPs) are channel-forming integral membrane proteins of the major intrinsic protein (MIP) family that allow the water transport across the cell membranes (King et al., 2004; Soveral et al., 2017; Yang, 2017). The secondary structure of AQPs consists of six transmembrane α-helices and two highly conserved, hydrophobic asparagine-proline-alanine (NPA) consensus motifs (Jung et al., 1994). The three-dimensional structure of AQPs resembles an hourglass with the NPA motifs forming the aperture of a very tight water channel pore of ~2 Å in diameter. In the cellular membranes, AQPs exist as a tetrameric assembly of individually active subunits. Professor Peter Agre was awarded the 2003 Nobel Prize in Chemistry for the discovery and characterization of the first water channel protein AQP1 (Agre, 2009). To date, thirteen aquaporins have been discovered (AQP0-AQP12) in mammalian tissues, which are classified into three subgroups depending on their permeability and structure: orthodox aquaporins, aquaglyceroporins and superaquaporins (Verkman et al., 2014). Orthodox aquaporins (AQP0, 1, 2, 4, 5, 6, and 8) are considered pure water channels, whereas aquaglyceroporins (AQP3, 7, 9, and 10) are permeated by water and other small solutes, such as glycerol or urea (Oliva et al., 2010). Superaquaporins (AQP11 and 12) exhibit very low homology to the other groups of AQPs due to their unique asparagine-proline-cysteine (NPC) motifs (Soveral et al., 2017) and their subcellular localization on the membrane of intracellular organelles instead of the plasma membrane (Ishibashi, 2006; Calvanese et al., 2013).

The human *AQP7* gene, mapped to chromosome 9p13.3, was cloned from the adipose tissue in 1997 (originally named AQPap) (Ishibashi et al., 1997, 1998). The glycerol channel AQP7 plays a crucial role in the control of triacylglycerols (TG) accumulation and glucose homeostasis with *Aqp7*-KO mice exhibiting adult-onset obesity, impaired insulin secretion and insulin resistance (Maeda et al., 2004; Hara-Chikuma et al., 2005; Hibuse et al., 2005; Matsumura et al., 2007). AQP7 is markedly increased during adipocyte differentiation, because the *AQP7* gene promoter contains putative response elements for peroxisome proliferator-activated receptor α and γ (PPARα and PPARγ), the master transcription factor of adipogenesis (Kishida et al., 2001; Walker et al., 2007; Méndez-Giménez et al., 2015). In this sense, the administration of the PPARγ agonists rosiglitazone or pioglitazone, which are insulin-sensitizing drugs, to rodents has been shown to upregulate AQP7 expression in the adipose tissue (Kishida et al., 2001; Lee et al., 2005; Rodríguez et al., 2015b). Although AQP7 was considered the unique glycerol channel in human adipose tissue, AQP3, AQP5, AQP9, AQP10, and AQP11 also represent novel pathways for glycerol transport in human adipocytes (Frühbeck and Gómez-Ambrosi, 2001; Rodríguez et al., 2011b; Laforenza et al., 2013; Madeira et al., 2014b, 2015). In the basal state, perilipin-1 binds to AQP7 in the lipid droplets, thereby preventing localization of AQP7 to the plasma membrane where it can exert glycerol efflux activity (Hansen et al., 2016). In circumstances of negative energy balance, such as fasting or exercise, TG are hydrolyzed to glycerol and free fatty acids (FFA) by adipose triglyceride lipase (ATGL) as well as hormone-sensitive lipase (HSL) enzymes (Frühbeck et al., 2014; Méndez-Giménez, 2017). Both FFA and glycerol are released into the bloodstream and can be used as energy substrates in peripheral tissues. Several lipolytic stimuli, such as catecholamines, leptin, atrial natriuretic peptide, uroguanylin and guanylin, regulate the expression and translocation of aquaglyceroporins from the cytosolic fraction (AQP3) or the lipid droplets (AQP7) to the plasma membrane facilitating glycerol release from adipocytes (Kishida et al., 2000; Walker et al., 2007; Rodríguez et al., 2011b, 2015b, 2016). By contrast, lipogenic stimuli, such ghrelin and dexamethasone, downregulate the expression of AQP7 in adipocytes, which results in an increase in intracellular glycerol (Fasshauer et al., 2003; Rodríguez et al., 2009), a metabolite that induces changes in the conformation and enzymatic activity of glycerol kinase (GK), favoring the conversion of glycerol to glycerol-3-phosphate (Yeh et al., 2004). The consequent increase in glycerol-3-phosphate concentrations induces TG biosynthesis, leading to a progressive adipocyte hypertrophy (Hara-Chikuma et al., 2005). Noteworthy, the gene expression of the main lipogenic enzymes are downregulated in visceral adipose tissue of obese subjects (Ortega et al., 2010).

Circulating glycerol constitutes an important energy substrate during fasting with the liver being responsible for about 70–90% of whole-body glycerol metabolism (Reshef et al., 2003). AQP9 constitutes the main route for hepatocyte glycerol uptake (Jelen et al., 2011; Calamita et al., 2012), although the human liver also expresses the aquaglyceroporins AQP3, AQP7, and AQP10 (Rodríguez et al., 2014). AQP9 is mainly localized in the sinusoidal plasma membrane that faces the portal vein (Elkjaer et al., 2000; Nicchia et al., 2001; Gena et al., 2013; Rodríguez et al., 2014). In hepatocytes, glycerol is phosphorylated to glycerol-3-phosphate by GK, and glycerol-3-phosphate constitutes a precursor for hepatic gluconeogenesis as well as for the *de novo* TG synthesis (Rodríguez et al., 2014). The proportion of glycerol used for hepatic gluconeogenesis or lipogenesis mainly depends on the nutritional state (Kuriyama et al., 2002; Calamita et al., 2012), but a sexual dimorphism has been also observed in hepatocyte glycerol utilization (Nicchia et al., 2001; Lebeck et al., 2015; Rodríguez et al., 2015a). The close coordination between adipose and hepatic aquaglyceroporins is required for the control of whole-body glucose homeostasis as well as lipid accumulation in both rodents (Kuriyama et al., 2002; Rodríguez et al., 2015b) and humans (Catalán et al., 2008; Miranda et al., 2009; Rodríguez et al., 2011b).

The existence of several AQPs has been identified in the pancreas (Table 1), an exocrine and endocrine organ of the digestive system that plays an essential role in the onset of insulin resistance and type 2 diabetes (Delporte, 2014). Exocrine and endocrine cells account for 90 and 10%, respectively, of the total pancreatic cells. Exocrine cells comprise acinar cells, which synthesize and secrete digestive enzymes, and ductal cells that release most of the pancreatic juice. Endocrine cells are organized into small clusters of cells termed islets of Langerhans, which are composed by five cell subtypes, α-cells (20% of the total cells producing glucagon), β-cells (~70% producing insulin), δ-cells (10% producing somatostatin), polypeptide cells (5% producing PP) and ε-cells (<1% producing ghrelin). In the present review, we have focused on the role of AQPs in the physiology and pathophysiology of the pancreas, highlighting the role of pancreatic AQP7, which has emerged as a novel player in the control of β-cell function (Matsumura et al., 2007; Best et al., 2009; Louchami et al., 2012; Méndez-Giménez et al., 2017).

**Table 1:** Tissue distribution and biological function of pancreatic aquaporins.

**Type**	**Exocrine/endocrine pancreas**	**Cellular location**	**Biological function**	**References**
AQP0	Not detected (mice, rats and humans)	–	–	Isokpehi et al., 2009
AQP1	Exocrine pancreas (rat and humans)	Acinar cells, intercalated ducts and capillaries	Pancreatic fluid secretion	Koyama et al., 1997; Hurley et al., 2001; Ko et al., 2002; Burghardt et al., 2003
AQP2	Not detected (rats and humans)	–	–	Hurley et al., 2001; Isokpehi et al., 2009
AQP3	Exocrine pancreas (humans)	Acinar and collecting duct cells	Marker of tumor aggressiveness in pancreatic ductal adenocarcinomas	Ishibashi et al., 1995; Burghardt et al., 2003; Direito et al., 2017
AQP4	Negligible expression (rat and humans)	–	–	Koyama et al., 1997; Hurley et al., 2001; Burghardt et al., 2003
AQP5	Exocrine pancreas (humans)	Intercalated and intralobular ductal cells	Pancreatic fluid secretion and marker of tumor differentiation in pancreatic ductal adenocarcinomas	Burghardt et al., 2003; Direito et al., 2017
AQP6	Not detected (mice, rats and humans)	–	–	Isokpehi et al., 2009
AQP7	Endocrine pancreas (mice and rats)	β- and δ-cells	Control of insulin synthesis and secretion, triacylglycerol accumulation and proliferation of β-cells	Matsumura et al., 2007; Best et al., 2009; Méndez-Giménez et al., 2017
AQP8	Exocrine pancreas (rat and humans)	Acinar cells	Pancreatic fluid secretion	Koyama et al., 1997; Calamita et al., 2001; Hurley et al., 2001; Burghardt et al., 2003
AQP9	Not detected (rats and humans)	–	–	Isokpehi et al., 2009
AQP10	Not detected (humans)	–	–	Hatakeyama et al., 2001; Méndez-Giménez et al., 2017
AQP11	Negligible expression (humans)	–	–	Isokpehi et al., 2009
AQP12	Exocrine and endocrine pancreas (rats)	Acinar cells and β-cells	Maturation and exocytosis of zymogen granules, marker of pancreatic damage in acute pancreatitis and pancreatic steatosis	Itoh et al., 2005; Ohta et al., 2009; Méndez-Giménez et al., 2017

*AQP, aquaporin*.

## Aquaporins in the exocrine pancreas: regulation of pancreatic fluid secretion and markers of pancreatic damage

**Figure 1:**
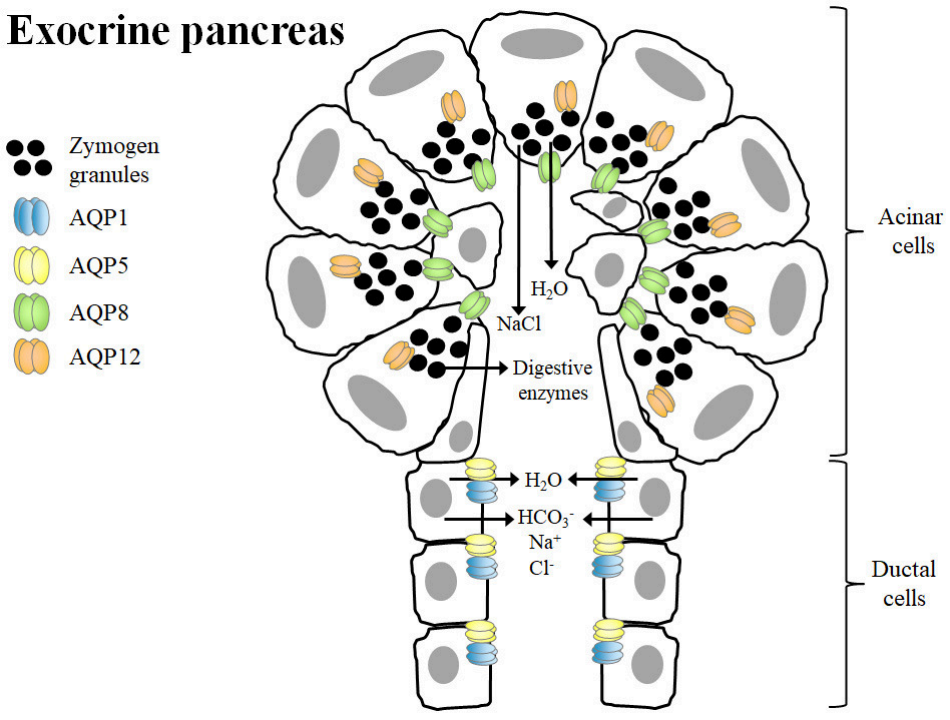
Role of aquaporins in isotonic fluid secretion and zymogen granule exocytosis in the exocrine pancreas. The primary function of pancreatic acinar cells is to synthesize and secrete digestive enzymes, which are stored in zymogen granules in the apical poles. AQP12, which is located in the cytoplasm, contributes to the proper formation, maturation and exocytosis of zymogen granules, a process dependent on water transport across the membranes. Acinar cells also secrete a small volume of a NaCl-enriched isotonic fluid. The water efflux from acinar cells to the lumen is mainly mediated by AQP8. Ductal cells secrete Na^+^, Cl^−^, HCO3^−^ as well as large amounts of water via AQP1 and AQP5 in order to form the final isotonic pancreatic fluid.

The exocrine pancreas secretes daily a large volume of ${\text{HCO}}_{3}^{-}$-rich fluid containing digestive enzymes to neutralize gastric acid that enters into the duodenum and to digest dietary nutrients (Hurley et al., 2001). The epithelial cells lining the ductal system and, to a lesser extent, acinar cells of the exocrine pancreas generate near-isotonic fluids, a mechanism that requires a high transepithelial water permeability (Hurley et al., 2001; Ko et al., 2002; Burghardt et al., 2003). Several AQPs, including AQP1, AQP5, AQP8, and AQP12, contribute to the high water permeability of apical and basolateral membranes of both acinar and ductal cells of the exocrine pancreas (Table 1). However, it is tempting to speculate that the existence of other unidentified water channels in the exocrine pancreas, since HgCl_2_, a non-selective AQP blocker, reduces total water permeability by as much as 90% in isolated rat acinar cells (Hurley et al., 2001) and 78% in isolated rat interlobular ducts (Ko et al., 2002). In the human pancreas, AQP8 is exclusively expressed in the apical membrane of pancreatic acinar cells, whereas AQP1 and AQP5 are abundantly expressed in the apical and basolateral membranes of the epithelial cells of intercalated ducts, which is probably the main site of pancreatic fluid secretion (Burghardt et al., 2003; Figure 1). In this sense, pancreatic fluid secretion starts with the secretion of a small volume of isotonic-like fluid rich in NaCl from acinar cells with AQP8 allowing water efflux to the lumen. Subsequently, intercalated ducts, secrete Na^+^, HCO3^−^, and Cl^−^ with AQP1 and AQP5 allowing water movement from ductal cells to the ductal lumen (Delporte, 2014). The digestive enzymes within the pancreatic juice are synthesized in acinar cells and are stored in secretory vesicles termed zymogen granules in the apical pole of the cell. Interestingly, AQP12 is expressed in the pancreatic acinar cells with an intracellular localization in the rough endoplasmic reticulum and the membranes of zymogen granules near the rough endoplasmic reticulum (Itoh et al., 2005; Figure 1). AQP12 not only participates in the secretion of pancreatic isotonic fluid, but it is also involved in the proper maturation and exocytosis of the zymogen granules in murine pancreatic acinar cells (Itoh et al., 2005; Ohta et al., 2009). There is evidence of expression of AQP12 in the human pancreas (Isokpehi et al., 2009), and its tissue distribution and function warrants further investigation.

The pancreatic exocrine function is severely impaired during pancreatitis, the inflammation of the pancreas, which is divided in acute and chronic types (Delporte, 2014; Ravi Kanth and Nageshwar Reddy, 2014). The clinical symptoms of acute pancreatitis include upper abdominal pain, nausea, vomiting and increased serum levels of the digestive enzymes amylase and lipase. Chronic pancreatitis is characterized by recurrent abdominal pain, damage of the pancreatic parenchyma with inflammation and fibrosis, ductal dilation, necrosis and finally a progressive loss of exocrine (maldigestion) and endocrine (diabetes mellitus) functions. Changes in both AQP1 and AQP12 have been observed during the onset of acute and chronic pancreatitis, reflecting their potential as markers of pancreatic damage. AQP1 is overexpressed in the pancreatic ducts of patients with autoimmune pancreatitis, which showed chronic pancreatitis characterized by a severely impaired secretion of digestive enzymes from acinar cells as well as pancreatic fluid and ${\text{HCO}}_{3}^{-}$ secretion from ducts (Ko et al., 2009; Koyama et al., 2010). The upregulation of AQP1 might constitute a compensatory mechanism to overcome the slowed fluid movement across the pancreatic endothelia and ducts, which alter the convective flow of pancreatic digestive enzymes through the pancreatic duct. On the other hand, AQP12 deficiency increases the susceptibility of caerulein, a cholecystokinin-8 analog inducing acute pancreatitis (Ohta et al., 2009). Accordingly, AQP12-KO mice show more numerous and larger exocytic vacuoles in acinar cells, an important cellular hallmark of early pancreatitis, than control mice.

Likewise, a deregulation of AQPs has been also detected in other pathophysiological conditions of the pancreas, such as obesity-associated pancreatic steatosis (Méndez-Giménez et al., 2017) or pancreatic ductal adenocarcinoma (Direito et al., 2017). AQP12 is upregulated in the pancreas of obese rats with the increased pancreatic *Aqp12* mRNA levels being positively associated with markers of insulin resistance and ectopic lipid overload (Méndez-Giménez et al., 2017). A strong immunoreactivity for AQP3 and AQP5 is observed in the ductal cells of patients with pancreatic ductal adenocarcinomas that is associated to tumor aggressiveness and tumor differentiation, respectively (Direito et al., 2017).

## Aquaporin-7 in the endocrine pancreas: control of insulin release, triacylglycerol accumulation and β-cell proliferation

**Figure 2:**
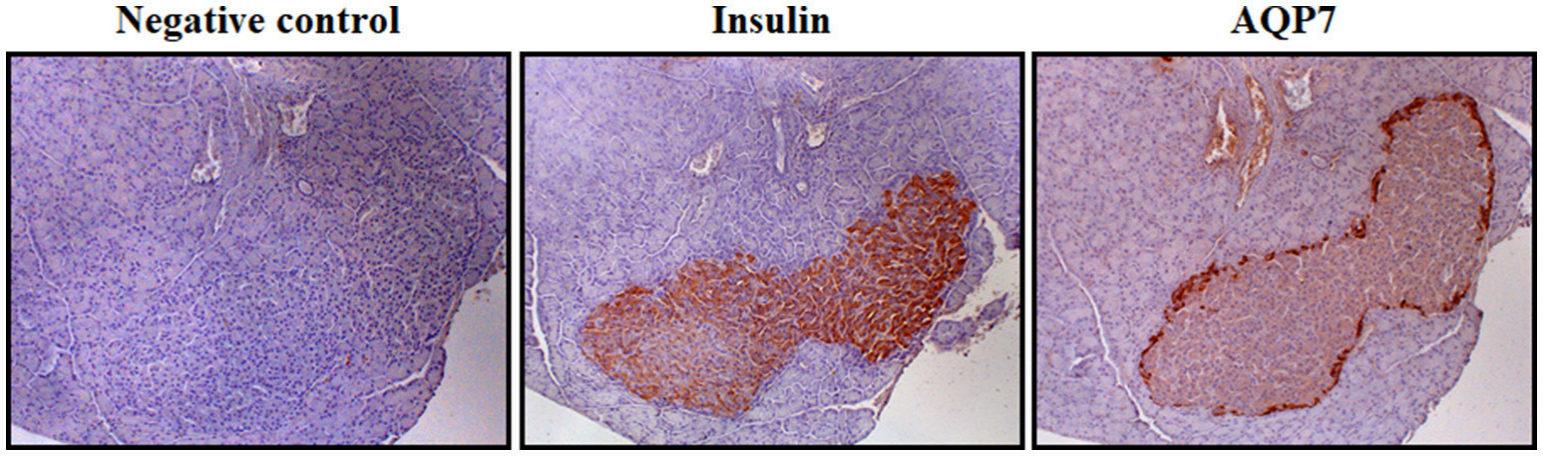
AQP7 distribution in β-cells of Langerhans islets. Immunohistochemistry showing the location of insulin and AQP7 in Langerhans islets in serial sections of rat pancreas using specific primary antibodies (magnification 100x). Negative control was obtained in the absence of primary antibody. The detailed methodology is described in the following reference (Méndez-Giménez et al., 2017).

**Figure 3:**
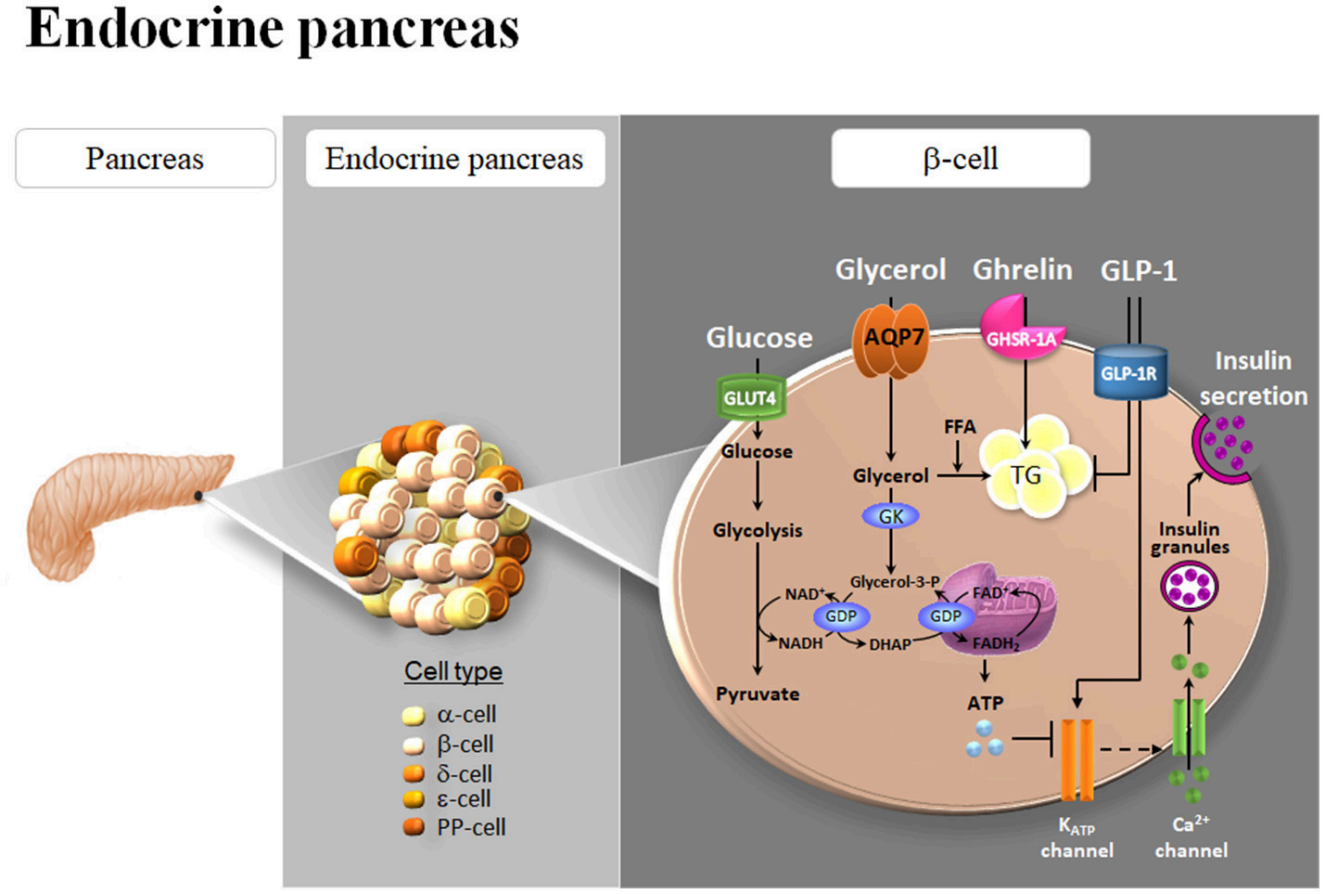
Role of AQP7 in insulin secretion and triacylglycerol accumulation in β-cells of the endocrine pancreas. AQP7 facilitates glycerol influx to β-cells. The increase in intracellular glycerol and the consequent activation of glycerol kinase (GK) activity, in turn, stimulate the pro-insulin mRNA and insulin secretion, probably through their participation in the glycolysis and glycerol-phosphate shuttle activities in the β-cell. Glycerol can be also used as a substrate for *de novo* synthesis of TG. Both ghrelin and glucagon-like peptide 1 (GLP-1) down-regulate AQP7 expression in β-cells. The subsequent increase in intracellular glycerol might be used for the biosynthesis of triacylglycerols (TG) induced by ghrelin as well as for insulin synthesis and secretion triggered by GLP-1. GDP, glycerol-3-phosphate dehydrogenase; GLUT4, glucose transporter 4; PP, pancreatic polypeptide.

Circulating glucose is the most relevant regulator of proinsulin synthesis and insulin secretion in pancreatic β-cells (Muoio and Newgard, 2008). However, glycerol constitutes another metabolite involved in nutrient-induced insulin release through the activation of the glycerol-phosphate shuttle, a metabolic pathway that replenishes cytosolic NAD^+^ levels necessary to maintain glycolysis, which in turn provides pyruvate for anaplerosis (Skelly et al., 2001). AQP7 has been identified in pancreatic β-cells of murine and rat endocrine pancreas (Figure 2), but not in the acini or the ducts of the exocrine pancreas, as well as in the rat pancreatic BRIN-BD11 and RIN-m5F β-cell lines (Matsumura et al., 2007; Best et al., 2009; Delporte et al., 2009; Méndez-Giménez et al., 2017). AQP7 transports urea and glycerol in β-cells, resulting in a similar β-cell swelling, activation of the volume-regulated anion channel and insulin secretion (Best et al., 2009). Nonetheless, glycerol triggers a more marked and sustained effect on membrane potential (Best et al., 2009). Extracellular glycerol is transported into β-cells through AQP7, transformed into glycerol-3-phosphate by the activation of the GK enzyme activity and entered into the glycerol-3-phosphate shuttle (Rodríguez et al., 2011a; Figure 3). In this metabolic process, glycerol-3-phosphate is converted into dihydroxyacetone phosphate (DHAP) in a reaction catalyzed by the inner membrane-bound mitochondrial glycerol-3-phosphate dehydrogenase (GPD) that reduces FAD^+^ to FADH_2_ that enters in the mitochondrial oxidative phosphorylation process to generate ATP (Skelly et al., 2001; Matsumura et al., 2007; Rodríguez et al., 2011a; Méndez-Giménez, 2017). The subsequent increase in the cytosolic ATP/ADP ratio induces the closure of ATP-sensitive K^+^ channels, depolarization of the plasma membrane, activation of voltage-dependent Ca^2+^ channels followed by a rapid influx of Ca^2+^ that triggers insulin exocytosis. *Aqp7*-KO mice show increased β-cell glycerol content and GK activity, which result in higher basal and glucose-induced insulin secretion (Matsumura et al., 2007; Louchami et al., 2012). Interestingly, AQP7 deficiency is associated with reduced β-cell mass caused by a decrease in β-cell proliferation, but it is also related to increased insulin-1 and insulin-2 transcript levels indicating a more efficient insulin biosynthesis and secretion (Matsumura et al., 2007).

Short-term exposure of β-cells to FFA increases glucose-stimulated insulin secretion, but chronic exposure to high FFA levels promotes β-cell hypertrophy and insulin hypersecretion, ultimately causing β-cell dysfunction and death through lipoapoptosis (El-Assaad et al., 2003; Méndez-Giménez et al., 2017). β-cells require a continuous sense fuel load, particularly glucose, and they cannot protect themselves by blocking glucose uptake to avoid excess nutrient load and their capacity to store fuel excess in the form of TG is limited (Mugabo et al., 2017). Excess-fuel detoxification pathways comprise glycerol and FFA formation and release to the extracellular milieu and the diversion of glucose carbons to TG and cholesterol esters in β-cells. In this regard, AQP7 plays an important role in the modulation of intraislet glycerol concentration and TG synthesis (Figure 3; Matsumura et al., 2007; Louchami et al., 2012; Méndez-Giménez et al., 2017). AQP7 deficiency in mice causes an increased intracellular glycerol and the GK activity resulting in an increase in TG concentration in the Langerhans islets (Hibuse et al., 2005; Matsumura et al., 2007).

## Obesity is associated with deregulation of aquaglyceroporins in the adipose tissue, liver and pancreas

AQP7-KO mice display a clear phenotype of adult-onset obesity and hyperinsulinemia (Maeda et al., 2004; Hibuse et al., 2005; Matsumura et al., 2007), but the impact of AQP7 loss-of-function homozygous mutations in human obesity and diabetes remains controversial. In a study conducted in 160 adult Japanese subjects, those individuals carrying homozygous missense mutations (R12C, V59L, and G264V) and silent mutations (A103A and G250G) in the *AQP7* gene neither exhibit obesity nor diabetes (Kondo et al., 2002). Nonetheless, Kondo and colleagues found that the unique case homozygous for G264V mutation in the *AQP7* gene exhibited an impaired exercise-induced increase in plasma glycerol in spite of the increased plasma noradrenaline (Kondo et al., 2002), confirming the role of AQP7 in adipocyte lipolysis. Hyperglyceroluria and platelet secretion defect have been also attributed to the G264V variant of *AQP7* gene in three children homozygous for this non-functional mutation (Goubau et al., 2013). In another cohort of 178 Caucasian subjects, one single case of a subject homozygous for the G264V mutation exhibited glycerol levels below the 10th percentile, overweight and type 2 diabetes (Ceperuelo-Mallafré et al., 2007). Moreover, a study performed in 977 Caucasian individuals detected a single-nucleotide polymorphism (A593G) in the human *AQP7* gene promoter that was related to decreased AQP7 expression in the adipose tissue as well as with type 2 diabetes (Prudente et al., 2007). Further studies are required to analyze the real impact of *AQP7* gene variants in the onset of obesity and type 2 diabetes. Nonetheless, growing evidence support the strong metabolic impact of the regulation of AQP7 expression in the onset of obesity and its associated comorbidities (Frühbeck, 2005; Frühbeck et al., 2006; Méndez-Giménez et al., 2014). Human obesity is associated with a deregulation in the expression of aquaglyceroporins in adipose tissue (Marrades et al., 2006; Ceperuelo-Mallafré et al., 2007; Prudente et al., 2007; Catalán et al., 2008; Rodríguez et al., 2011b) and liver (Catalán et al., 2008; Miranda et al., 2009; Rodríguez et al., 2014). Visceral adipose tissue of obese patients shows an upregulation of AQP3 and AQP7, which might be related to the increased lipolytic rate in this fat depot (Catalán et al., 2008; Rodríguez et al., 2011b). In contrast, AQP7 is downregulated in the subcutaneous adipose tissue leading to the promotion of an intracellular glycerol accumulation and a progressive adipocyte hypertrophy (Rodríguez et al., 2011b). Moreover, our group found a reduction of glycerol permeability and AQP9 expression in the liver of obese patients with non-alcoholic fatty liver disease (NAFLD) in parallel to the degree of hepatic steatosis, being further aggravated in insulin-resistant patients (Rodríguez et al., 2014). The downregulation of AQP9 seems to be a compensatory mechanism whereby the liver counteracts further TG accumulation within its parenchyma as well as reduces hepatic gluconeogenesis in obese patients with NAFLD.

Obesity is commonly associated with insulin resistance and type 2 diabetes. Under normal conditions, the pancreatic islet β-cells increase insulin secretion sufficiently to overcome the reduced efficiency of insulin action, thereby maintaining normal glucose tolerance. In order to maintain an appropriate long-term glycemic control in insulin-resistant states, the number of pancreatic islet β-cells or β-cell mass, is expanded (de Koning et al., 2008; Méndez-Giménez, 2017). The β-cell dysfunction is characterized by a decreased insulin gene expression, blunted glucose-stimulated insulin secretion as well as increased β-cell apoptosis rates (Wajchenberg, 2007). Obesity-associated insulin resistance has been attributed to ectopic lipid overload, with lipotoxicity being a major contributor of β-cell dysfunction (Lee et al., 2010; van Raalte et al., 2010; Ou et al., 2013). Since glycerol also constitutes an important metabolite for insulin exocytosis and TG synthesis in β-cells, we analyzed the impact of obesity and weight loss achieved by bariatric surgery on pancreatic AQP7 in a recent study (Méndez-Giménez et al., 2017). As expected hyperinsulinemic and insulin-resistant obese rats exhibited adaptive changes in β-cell mass as well as pancreatic steatosis. Bariatric surgery improved β-cell dysfunction in obese rats, as evidenced by reduced pancreatic β-cell apoptosis, steatosis and insulin secretion. Interestingly, both weight gain and weight loss achieved by bariatric surgery were associated with increased pancreatic AQP7 mRNA and protein levels (Méndez-Giménez et al., 2017). AQP7 upregulation in the pancreas might constitute an adaptive response of β-cells to increase glycerol uptake and the subsequent insulin synthesis and secretion, which seems nevertheless inefficient to reduce the hyperglycemia in the obese state, but not after bariatric surgery. Further studies are needed to validate the potential role of AQP7 in β-cell function in the human pancreas.

## Role of AQP7 in ghrelin- and GLP-1-induced improvement of pancreatic β-cell function after bariatric surgery

Bariatric surgery significantly improves insulin sensitivity within days after this procedure, which implicates mechanisms independent of weight loss that involve the modulation of intrinsic gut hormones via the gastro-entero-insular axis (Frühbeck, 2015; Méndez-Giménez, 2017). The incretin hormone glucagon-like peptide-1 (GLP-1) is among the most widely studied modulators of β-cell function, with the incretin effect accounting for 70% of the insulin secretion after an oral glucose tolerance test (Hussain et al., 2016). At the endocrine pancreas, GLP-1 binds its receptor GLP-1R and suppresses glucagon secretion from α-cells and potentiates insulin secretion from β-cells in a glucose-dependent manner. On the other hand, ghrelin represents a survival factor promoting cell survival *in vitro* in HIT-T15 pancreatic β-cells (Granata et al., 2006) and *in vivo* in streptozotocin-induced diabetic mice (Bando et al., 2013). Interestingly, we found that acylated and desacyl ghrelin induced intracellular lipid accumulation in RIN-m5F β-cells (Méndez-Giménez et al., 2017), which is in agreement with the lipogenic effect of ghrelin isoforms in other metabolic tissues, including adipose tissue and liver (Rodríguez et al., 2009; Porteiro et al., 2013; Ezquerro et al., 2016). We confirmed the water (*Pf*) and glycerol (*Pgly*) permeability of the rat RIN-m5F β-cells (Méndez-Giménez et al., 2017), which exhibited permeability values within the range of the *Pf* and *Pgly* measured in mature murine 3T3-L1 adipocytes with endogenous AQP7 expression (Madeira et al., 2013). To gain further insight into the molecular mechanisms triggering the improvement of β-cell function, the role of ghrelin and GLP-1 in the expression of pancreatic AQP7 was studied (Figure 3). Acylated and desacyl ghrelin constitute negative regulators of AQP7 in adipocytes and this downregulation contributes, in part, to the lipid accumulation in fat cells (Rodríguez et al., 2009). Accordingly, acylated and desacyl ghrelin diminished the AQP7 expression in parallel to an increased TG content in RIN-m5F β-cells (Méndez-Giménez et al., 2017). Interestingly, GLP-1 showed a tendency toward a downregulation of AQP7 in RIN-m5F β-cells with the AQP7 protein expression being negatively associated with insulin release (Méndez-Giménez et al., 2017). Thus, it seems plausible that the reduction of AQP7 induced by ghrelin and GLP-1 might result in intracellular glycerol accumulation, which can be used for the biosynthesis of TG as well as for insulin synthesis and secretion in β-cells (Figure 3).

## Conclusions

Although there is compelling evidence from murine and human studies that AQP7 might constitute an effective anti-diabetic drug target (Rodríguez et al., 2011a; Méndez-Giménez et al., 2014; da Silva and Soveral, 2017), the discovery and development of pharmacological AQP modulators has been slow, in part because current efforts to identify inhibitors are hampered by challenges in screening assays and in targeting the compact, pore-containing AQP molecule (Verkman et al., 2014).

Several heavy metals, such as mercury chloride (HgCl_2_), silver sulfide (AgS) or gold(III) compound [Au(phen)Cl_2_]Cl (phen = 1,10-phenatroline) (Auphen) constitute AQP7 inhibitors (Preston et al., 1993; Delporte et al., 2009; Madeira et al., 2014a). Hg^2+^ ions bind specifically the mercury-sensitive cysteine located just in front of the second NPA box of several AQPs (Preston et al., 1993). This covalent modification of cysteine residues either induces the blockage or conformational change of the AQP pore causing the inhibition of water permeability. Silver (AgNO_3_ or AgS) or gold (HAuCl_4_ or Auphen) compounds interact with sulfhydryl groups of proteins, such as the thiolates of cysteine residues in the vicinity of conserved NPA motifs and thus effectively inhibit water and glycerol permeability to a higher extent than HgCl_2_ (Niemietz and Tyerman, 2002; Martins et al., 2012). However, the AQP inhibitors HgCl_2_ or AgS cause major irreversible cytotoxic effects in β-cells (Best et al., 2009). In this context, the design of novel small-molecule modulators of AQP7 expression/function may have clinical applications in the therapy of type 2 diabetes. The use of well-designed experimental strategies is of utmost importance for aquaporin drug discovery. The most frequently used biophysical and biological approaches to detect AQP activity and/or function include: (i) cell models with AQP gene overexpression or silencing for functional analysis; (ii) water and/or glycerol permeability assays by using techniques based on volume-dependent optical properties, such as stopped-flow light-scattering spectrophotometry, or osmotic swelling assays; and (iii) computational methods for the analysis of the target of novel candidate molecules in the three-dimensional AQP structure models (for extensive review of these methods, please refer to; Madeira et al., 2016).

From a clinical point of view, the possibility of regulating the pancreatic AQP7 function, by the upregulation of AQP expression or possibly by gene transfer in rare cases of loss-of-function mutations of the *AQP7* gene, may also be beneficial in obesity, insulin resistance and type 2 diabetes. Nonetheless, additional data related to novel mutations, single nucleotide polymorphisms, epigenetic and transcription changes and protein stability are needed to better establish a firm mechanistic basis for the contribution of AQP7 in the etiopathogenesis of these metabolic diseases.

## Author contributions

AR: Conception and design of research; LM-G, GF, and AR: Prepared figures; LM-G, GF, and AR: Drafted the manuscript; LM-G, SE, IdS, GS, GF, and AR: Edited and revised manuscript; LM-G, SE, IdS, GS, GF, and AR: Approved final version of manuscript.

### Conflict of interest statement

The authors declare that the research was conducted in the absence of any commercial or financial relationships that could be construed as a potential conflict of interest.

## Abbreviations

AQP: aquaporin
ATGL: adipose triglyceride lipase
DHAP: dihydroxyacetone phosphate
GK: glycerol kinase
GLP-1: glucagon-like peptide 1
GPD: glycerol-3-phosphate dehydrogenase
FFA: free fatty acids
HSL: hormone-sensitive lipase
NAFLD: non-alcoholic fatty liver disease
PPAR: peroxisome proliferator-activated receptor
TG: triacylglycerols.
